# Maternal HIV disclosure to HIV-uninfected children in rural South Africa: a pilot study of a family-based intervention

**DOI:** 10.1186/1471-2458-13-147

**Published:** 2013-02-18

**Authors:** Tamsen J Rochat, Ntombizodumo Mkwanazi, Ruth Bland

**Affiliations:** 1 Africa Centre for Health and Population Studies, University of KwaZulu-Natal, Mtubatuba, South Africa, and Department of Psychology, Stellenbosch University, Stellenbosch, South Africa; 2 Africa Centre for Health and Population Studies, University of KwaZulu-Natal, Mtubatuba, South Africa, and School of Public Health, University of Witwatersrand, Johannesburg, South Africa; 3 Africa Centre for Health and Population Studies, University of KwaZulu-Natal, Mtubatuba, South Africa; School of Medicine, University of Glasgow, Glasgow, United Kingdom, and School of Public Health, University of Witwatersrand, Johannesburg, South Africa

**Keywords:** Intervention, HIV-disclosure, Family-centred, Maternal HIV-disclosure

## Abstract

**Background:**

As access to treatment increases, large numbers of HIV-positive parents are raising HIV-negative children. Maternal HIV disclosure has been shown to have benefits for mothers and children, however, disclosure rates remain low with between 30-45% of mothers reporting HIV disclosure to their children in both observational and intervention studies. Disclosure of HIV status by parent to an HIV-uninfected child is a complex and challenging psychological and social process. No intervention studies have been designed and tested in Southern Africa to support HIV-positive parents to disclose their status, despite this region being one of the most heavily affected by the HIV epidemic.

**Method:**

This paper describes the development of a family-centred, structured intervention to support mothers to disclose their HIV status to their HIV-negative school-aged children in rural South Africa, an area with high HIV prevalence. The intervention package includes printed materials, therapeutic tools and child-friendly activities and games to support age-appropriate maternal HIV disclosure, and has three main aims: (1) to benefit family relationships by increasing maternal HIV disclosure; (2) to increase children’s knowledge about HIV and health; (3) to improve the quality of custody planning for children with HIV-positive mothers. We provide the theoretical framework for the intervention design and report the results of a small pilot study undertaken to test its acceptability in the local context.

**Results:**

The intervention was piloted with 24 Zulu families, all mothers were HIV-positive and had an HIV-negative child aged 6–9 years. Lay counsellors delivered the six session intervention over a six to eight week period. Qualitative data were collected on the acceptability, feasibility and the effectiveness of the intervention in increasing disclosure, health promotion and custody planning. All mothers disclosed something to their children: 11/24 disclosed fully using the words "HIV" while 13/24 disclosed partially using the word "virus".

**Conclusion:**

The pilot study found the intervention was feasible and acceptable to mothers and counsellors, and provides preliminary evidence that participation in the intervention encouraged disclosure and health promotion. The pilot methodology and small sample size has limitations and further research is required to test the potential of this intervention. A larger demonstration project with 300 families is currently underway.

## Background

Prevention of Mother-to-Child HIV Transmission (PMTCT) programs in sub-Saharan Africa have been extremely successful in reducing the numbers of children infected with HIV [1]. In South Africa with its extensive use of effective PMTCT regimens new HIV infections in infants in the future should be rare [2,3]. However, in high HIV prevalence areas, at least 40% of pregnant women may be HIV-infected, and as a consequence large numbers of HIV-uninfected children are being cared for by at least one HIV-infected parent [4]. HIV-infected mothers face the dilemma of when, and how, to disclose their own HIV status to their children [5-7].

Research, mainly from the United States of America (USA) and Europe, has shown that maternal HIV disclosure has benefits for mothers and children [8-10], including improvements in maternal mental health [11-14] and the quality of relationship between mother and child [9,10], increased preparation for the child’s future in terms of custody and care planning for emergencies [8,15,16], and improvements in children’s emotional and social functioning [16,17]. However, disclosure rates remain low with between 30-45% of mothers reporting HIV disclosure to their children in both observational and intervention studies [5-7,18,19]. Only a few studies have explored paternal HIV disclosure [20-23], and most intervention research has focused on mothers disclosing to their children. While the reasons for this are likely pragmatic since the majority of HIV-affected children live with, and are cared for, primarily by their mothers, this exclusion of fathers (whether biological or social) is increasingly critiqued in the literature on family interventions in the context of HIV [24].

There is limited research relating to maternal HIV disclosure in the African context [7], and few studies globally have targeted mothers with primary school-aged children [9,25-27]. Only one recent intervention study from the United States (US) evaluated a maternal HIV disclosure intervention (the TRACK program) targeted at primary school-aged children [28], demonstrating that disclosure interventions in pre-adolescence are not only feasible, but also effective among mothers who had no intention to disclose to their children at the start of the study. TRACK was a randomized controlled trial of an intervention which included three home visits, telephone support and educational materials. Intervention mothers were over four times more likely to disclose, with a disclosure rate of 33% in the intervention compared to 7.3% in the control arm.

More generally in the literature, mothers frequently express the desire to disclose their HIV status to their children themselves, but report feeling unsure about how to approach the issue [18,19,29,30]; are worried about addressing matters concerning sex and sexuality [15,31,32]; and have concerns that disclosure may cause emotional difficulties for their child [18,19,29]. A significant amount of international research has been conducted on the consequences of a lack of maternal HIV disclosure among 6–18 year olds [33]. While few mothers report disclosing to their children in the primary school years many are forced to disclose before adolescence following HIV-related illnesses or hospitalisations [21], which impacts negatively on children’s emotional and behaviour outcomes in case–control studies [5-7]. In most disclosure research to date mothers emphasize the need for assistance in planning and preparing towards disclosure [5,19,29,31,32], an important consideration in the design of disclosure interventions for Southern Africa.

In considering approaches to maternal HIV disclosure in the Southern African context, unique challenges exist. Although the largest population of HIV-infected parents lives in Africa, very little research has examined maternal HIV disclosure to children in this region. As access to HIV treatment increases, so does exposure to HIV at a family level. For example, by January 2010, 3% of the population in the research area had initiated ART, and as a result 40% of the population shared household or living arrangements with people who had either started ART or were enrolled into pre-ART care [34]. Therefore, children living in communities where ART is being scaled up are more likely to be socially exposed to HIV at younger ages. In general, increases in access to treatment also tend to reduce stigma within communities and families, allowing for more preventive activities at a household and family level [35].

Concurrently, there is growing evidence of the risks facing pre-adolescent and adolescent children growing up in HIV-affected families in Southern Africa [36-38]. Therefore, disclosure interventions that target younger children in these contexts may have the benefit of helping not only with parental disclosure but also of promoting more generalised preventive interventions including improved quality of parent–child relationships, health promotion and custody planning. Given that South Africa has the largest antiretroviral treatment (ART) programme in the world there are unique opportunities to explore psychological interventions, using family approaches, to develop culturally-appropriate disclosure interventions. These interventions should draw on existing evidence, and be feasible for integration into standard of care psychosocial support for HIV-infected parents, if found to be effective. We describe here the development and piloting of a complex psychological intervention (the ‘Amagugu’ ^a^ Intervention) to support age-appropriate maternal HIV disclosure to primary school-aged children, aged 6–10 years, to be delivered by lay counsellors or community health care workers in poorly resourced settings.

## Methods

### The research context

The Wellcome Trust-funded Africa Centre for Health and Population Studies (http://www.africacentre.com) is based in rural northern KwaZulu-Natal, South Africa. HIV prevalence in 2010, within the Africa Centre Demographic Surveillance Area (DSA), that includes 90,000 adults, was 23% among adults overall [39]. Over the period 2005–2010 the estimated annual incidence was about 3% in adults of all ages, reaching a high of nearly 7.5% in females aged 25–29 years old and over 5% in men 30–34 years old [40]. Whilst HIV prevalence is increasing due to the widespread availability of antiretroviral treatment (ART) there is no evidence of a declining HIV incidence since measurement started in 2003 [41]. The demographic profile of the study area shows that the majority of children live with their biological mothers, not with their fathers [42,43]. This intervention was therefore designed primarily for mothers, but is flexible in its inclusion of fathers as described below.

### The intervention aim

The intervention package was developed to include printed materials, therapeutic tools and child-friendly activities and games to support age-appropriate maternal HIV disclosure. It has three main aims:

(1) to benefit family relationships by increasing maternal HIV disclosure;

(2) to increase children’s knowledge about HIV and health;

(3) to improve the quality of custody planning for children with HIV-positive mothers.

### Evidence base and theoretical framework for the intervention design

In line with the UK Medical Research Council revised guidelines for developing complex interventions [44] the Amagugu design is based on a comprehensive review of the evidence base and a clear theoretical understanding of what is needed to effect change in disclosure interventions, informed by what we know about children's development at this age and the cultural context.

A literature review on maternal HIV disclosure to children was conducted, finding a total of 58 studies, including two literature reviews [5,6] and a recent systematic review [7], all of which are reported on, and summarised, by the authors in a recently published Special Report [33]. In addition we reviewed the recent guidelines from the World Health Organization on HIV disclosure to children [45] that include the available evidence on maternal disclosure to HIV-negative children of primary school-age and highlight the lack of studies on this issue.

The Amagugu design builds on a theoretical model developed from previous work [28,46] and contains six steps as outlined in Table 1. The intervention is designed to provide the mother with the opportunity to receive information, and to plan and practise a series of intervention activities to be able to facilitate safe, developmentally appropriate, disclosure with her child. Fathers and other family members are encouraged to participate in activities, and the intervention can be used by other care-givers of the child. The HIV education materials developed for Session 4, 5 and 6 (see Table 1) also have the potential to be adapted for use with HIV-infected children in this age range, although this does not fall within the scope of the current study.

**Table 1:** The Amagugu intervention detailing the six steps, purpose of the steps, materials and activities and session goals

**Session/Visit**	**Description**	**Purpose**	**Materials and activities**	**Session goals**
1: Positive parenting	*One-on-one counselling session between mother and lay counsellor which takes place by arrangement at the home*	Assess mothers' readiness to disclose, offer psycho-education on disclosure steps, communicate pros and cons of disclosure, encourage an informed decision to participate and proceed with disclosure	**My intervention steps poster**	Increase mothers’ knowledge about age-appropriate disclosure
			*A3 illustrated colour poster*	
			**My intervention calendar**	Increase mothers' understanding of risks and benefits of disclosure
			*A5 illustrated colour card*	
2: Positive families	*Family visit co-facilitated by the counsellor and mother - family invited by the mother (either biological or socially-defined members); not an HIV specific activity as children are present*	Engage family members in a fun family process, create a sense of belonging and pride, use a strengths-based model, communicate respect for culture and values, introduce concept of play-for-communication and materials	**My family tree**	Increase family awareness of parenting aims of the intervention
			*A2 family tree poster with 14 illustrated sticker characters*	Encourage a sense of belonging among all family members
3: Positive life stories	*One-on-one counselling session between mother and counsellor using artwork and narrative therapy activities*	Improve emotional well-being of the mother in preparation for disclosure; use ‘head, hands and heart’ model to maintain empathy and introduce concepts of emotional work; use artwork to facilitate positive experiences of love, relationships and to unpack the story of becoming HIV infected and living with HIV; dispel myths and misperceptions about HIV and offer HIV information	**My life story**	Mother feels supported, respected and understood by the counsellor
			*A4 paper, artwork materials*	Mother constructs a family/parenting narrative
			**My HIV story**	Mother more emotionally contained and able to focus on the child
			*A4 illustrated HIV story telling exercise*	Mother has confidence in her HIV specific knowledge
4: Positive practices	*One-on-one training session to personalise 5-step disclosure intervention and practise using role-play*	Offer specific guidance on planning and preparing the disclosure event (time, place and people)	**Talk and play reminder**	Mother increases her understanding of what is age-appropriate and is confident to talk about HIV in a child-friendly way
			*A5 illustrated colour card*	
		Teach the 5- step process and what each step offers the child	**Family life line**	Mother develops and practises her own talking points, has a language to use in approaching disclosure
			*A4 card artwork and stickers*	
		Demonstrate the 5-step disclosure intervention and teach age-appropriate activities and games	**HIV body map**	Reduce mothers' anxiety about ‘dreaded questions’ by developing child friendly and age-appropriate answers
			*A4 HIV body map game with stickers*	
		Through practice ensure that mother is skilled, confident, and feels enabled to undertake disclosure using the intervention tools and materials.	**Playing cards**	Mother understands child’s needs for information, activity, reassurance and comfort
			*A7 10 matching pairs playing card set*	
		Help mother develop her own child and family personalised materials	**Safety hand**	Mother is confident to proceed with disclosure
			*A4 card and pens for artwork*	
		Allow for role playing, practice and development of personal talking points	**Family treasures story book**	
			*A5 illustrated 14 page English-Zulu story book*	
5: Positive planning	*One-on-one training session to train mother on health promotion activities and to prepare for a joint parent-child clinic visit*	Encourage mothers to move beyond disclosure and increase health promotion and the child’s access to a health support network and improve custody planning. Demonstrate clinic visit games and activities and encourage mother to take the child on a clinic visit, empower mother to advocate for her own and her child’s health care needs, counsel around fears and stigma	**Clinic visit reminder**	Mothers move beyond disclosure to health promotion and planning
			*A5 illustrated colour card*	
			**Name tag**	Mother is able to advocate for her and her children’s health rights
			*A8 name tag with beaded strap*	
			**Clinic check list**	Mother ensures that HIV treatment is demystified and personalised for the child
			*A4 “I-spy” game*	
			**My care circle (health)**	Health care environment becomes familiar
			*A4 illustrated card with health care contact information*	Increase child’s participation
				Child has increased understanding of healthy sexuality
6: Positive futures	*Family session facilitated by mother and attended by counsellor, provides mother and children opportunities to share intervention learning and achievements*	Reinforce play-for-communication, teach mothers basic skills for developmental play, provide positive reinforcement for emotional sharing, allows the child to participate, develop self efficacy and pride around intervention achievements	**Uthando doll**	Mothers have increased understanding about how children use play to communicate
			*Handmade ethnically appropriate doll*	
			**My care circle (custody)**	Children feel valued and rewarded for participation
			*A4 illustrated card with emergency care contact information*	

Once disclosure is achieved, the intervention encourages mothers to engage in health promotion activities including a clinic visit (Session 5); and to develop custody and care plans (Session 6). Importantly while the counsellor, either a lay counsellor or community health care worker (CHW), offers assistance and trains the mother towards disclosure, the mother undertakes disclosure with the child on her own. Similarly, the mother takes the child to the clinic independently, and completes a care plan and custody plan without the counsellor being present. This is to ensure parenting skills transference, self-efficacy and to build the mother’s confidence to deal with HIV issues with her child. This intervention has been carefully designed to achieve specific tasks, in a specific order, each session building on the one before. The intervention process is described in detail in a train-the-trainer modelled training manual and DVD.

Three important principles related to the Southern African context informed the design and development of this intervention:

#### Family-centred, mother-driven, approach

There is growing acknowledgement of the key role played by families in managing the burden of HIV care in Southern Africa [47,48]. In this intervention when we refer to ‘family’ we are referring to the concept of the family relationship *context* within which the child is being raised, and which is the focus of this disclosure intervention. Family is thus broadly and functionally defined, suggesting that family members who are in relationship with one another, who live together and function as family, for whatever reasons, create a shared social reality for the child that is linked to the care and development of children in the context of maternal HIV illness [49-51]. The focus on the family relationship context is based on the assumption that family-like relationships have greater emotional intensity than most other social relationships and thus provide significant leverage for influencing the day-to-day care and support of children [52].

This family-centred intervention approach, like many in the literature on chronic illness, is designed to address several concurrent issues and thus remain generalisable to epidemic settings, and to have value beyond disclosure alone. Some of the more general issues addressed through the intervention approach include:

Firstly, it focuses on strengthening family relationships through a specific family engagement process, as opposed to simply providing HIV education to the mother. This is important because improvements in the quality of the parent–child and family relationship have shown particular promise in improving outcomes in children and adolescents following maternal HIV disclosure [5-7].

Secondly, we consider the context of stigma and how this may have limited initial acceptance by the mother of her own HIV infection. In many epidemic contexts mothers may have received no prior counselling, we thus offer support and education to adjust to the changes that HIV brings within the family, attempting to reduce the social isolation, stress and worries of the HIV- positive parent [53,54].

Thirdly, it aims to help family members, and parents in particular, to prevent HIV from dominating family life and sacrificing normal family or parenting goals [52,55]. Promoting parental self-efficacy is important given the possible effects of living in stigmatized communities [56].

Lastly, the intervention approach intends to provide a new structure and focus for the family which is centred on parenting and quality care for children, with adjustments of roles and expectations to ensure optimal self-care of the HIV-positive mother, care of the child and health promotion at a family level [57,58].

#### Home-based model delivered by lay counsellors

The principles outlined above are important to the design of evidence-based family-centred HIV interventions; however, in Southern Africa public health resources are scarce and intervention approaches need to be inexpensive and feasible. In areas of high HIV prevalence clinics are overburdened, with lack of waiting areas or private rooms in which to counsel patients, and shortages of health care staff [59-61]. This intervention has, therefore, been designed to be conducted in the home setting, thus not burdening the health facilities, by lay counsellors or CHWs with no tertiary or formal health education, who will receive structured training to conduct this intervention [62]. The intervention materials were designed to achieve the goals of disclosure and to prepare the mother, emotionally, to undertake disclosure in a child-centred way. However, lay counsellors and CHWs also work in time-pressured, task-heavy, roles and compassion fatigue is commonplace [59-61]. Therefore, several of the activities, such as the My Life Line and My HIV Story exercises (see Table 1) were developed to serve the dual purpose of preparing the mother for disclosure and keeping the counsellor compassionate and attentive.

#### Structured processes and tools that are developmentally appropriate and build skills in the mother

This intervention targets mothers in South Africa, and a younger age group of children than most previous studies, with the exception of the TRACK program in the United States (28). Whilst there is a dearth of intervention evidence on maternal disclosure globally, there is a significant body of literature around children’s understanding of illness linked to their developmental stage [63], and in particular children’s developmental capacity to understand and engage with illnesses such as cancer and HIV [64]. Literature relating to maternal terminal illness, but not HIV, suggests that children benefit from being provided with illness-related information, explanations about what they might see and expect in the patient, reassurance that they will be looked after even in difficult circumstances; and comfort that being upset is ‘allowed’ and ‘normal’ under the circumstances [52,65,66].

We also know that how the disclosure event is executed can influence the child’s ability to cope with the information in the future [5,6]. Therefore, this intervention is designed to take a structured approach, similar to the approach of the TRACK program (28), and introduces specific guidance, training and planning for the mother towards the disclosure event, including the provision of child-friendly and developmentally appropriate learning materials for use as part of the intervention. The counsellor ‘trains’ the mother in a step-by-step approach, and practises with her the use of the materials and tools until she is competent and confident to use them [53]. This intervention is hinged on a *strengths-based model*, and counselling aims to deliver core communications to build the mothers’ confidence and self-efficacy as a ‘good’ parent, ensuring mothers feel understood and cared about, are reminded of their resilience and achievements, understand the important role they play in their children’s healthy development and have their confidence built through practising parenting skills.

### Evaluation and assessment

Data are collected at baseline, including details relating to the mother’s health, as the literature suggests that disclosure to children often occurs as a result of illness or hospitalisation; prior disclosure of HIV status to other adults as we expected issues related to stigma and a lack of disclosure at the family level might influence the feasibility and acceptability of this intervention; and mother’s literacy in English and Zulu as illiteracy may prove a barrier to the usability of the tools. At baseline, prior HIV disclosure to any or all children within the family, as well as the mother’s intentions to disclose, were not considered as inclusion or exclusion criteria as we aimed to be as inclusive as possible in this exploratory pilot study. However, detailed data were collected on prior disclosures with reasons for, and against, disclosure.

Maternal disclosure to the study child following participation in the study was collected at Visit 5. To be as inclusive as possible we considered all levels of disclosure including whether the disclosure was ‘partial’ (i.e. the mother explained that she has a ‘virus’ and what this does in her body) or ‘full’ (i.e. the mother explained that she has a virus called ‘HIV’ and what this does in her body). Open-ended qualitative measures around mother’s perceptions of the disclosure event and the materials, the child’s initial reactions to disclosure, and how the mother felt about the materials were also collected. If mothers chose not to disclose following the training, they were still encouraged to complete the health promotion and custody planning components of the intervention. Pre and post data collection, including qualitative measures, were completed for all mothers irrespective of the level of disclosure they achieved. Further data were collected at Visit 7 on whether the mother and child attended the health promotion clinic visit and their experiences of this, and whether a care plan was drawn up for the child. Data were also collected on the impact of the intervention at a family level.

### Pilot study

The intervention was piloted with 24 Zulu families within the Africa Centre Demographic Surveillance Area from November to December 2010. The mothers (all HIV-positive) and children (all HIV-negative) had been part of a previous Africa Centre study and had learned of their HIV status during their pregnancy with the study child, now aged 6–10 years [67]. To be eligible for inclusion in this study mothers had to be HIV-infected at the time of the original study, to have an HIV-uninfected child, to be currently resident in the study area and to be living with the study child, and to be in reasonable physical and mental health to complete the intervention activities. Where appropriate, mothers not already on HIV treatment were referred to the local HIV treatment and care programme for medical assessment. As previously noted, baseline disclosure to children or family members and intention to disclose to children or family members was not used as part of the eligibility screening in this pilot study.

A convenience sample of 24 mothers who all lived in one geographical peri-urban area was approached to facilitate completing the pilot field work in a reasonable time frame. A list of all available mothers from this area was produced, ordered by study ID from the original study, and a consecutive series of mothers were approached by telephone and home visits until 24 mothers were enrolled. A detailed description of the characteristics of participating mothers is included in Table 2.

**Table 2:** Maternal HIV health and prior HIV disclosure experience

**Study number**	**Maternal age at study enrolment (Years)**	**First HIV diagnosis (Year)**	**Currently on ART (Initiation date)**	**Most recent CD4 count**	**Participant perception of physical health**	**Current partner HIV status**	**Disclosed to current partner**
01	31	2003	No	186	Good	HIV positive	Yes
02	48	2003	No	500	Very good	Don’t know	Yes
03	30	2003	No	707	Excellent	Don’t know	Yes
04	31	2004	No	388	Good	HIV positive	Yes
05	29	2002	Yes (May 2010)	243	Good	Don’t know	Yes
06	31	2002	No	747	Good	Don’t know	Yes
07	27	2002	Yes (July 2010)	102	Good	No partner	No partner
08	40	2003	No	100	Good	HIV positive	No
09	39	2003	Yes (April 2010)	1	Good	HIV positive	Yes
10	32	2002	No	76	Very good	Don’t know	Yes
11	27	2003	No	380	Excellent	Don’t know	No
12	37	2003	No	Don’t know	Good	Don’t know	No
13	37	2003	No	568	Good	Don’t know	No
14	33	2004	Yes (Nov 2005)	509	Good	Don’t know	No
15	46	2002	Yes (Nov 2008)	429	Good	HIV positive	Yes
16	41	2003	No	325	Good	HIV negative	Yes
17	34	2002	No	500	Excellent	No partner	No partner
18	25	2003	Yes (Jan 2009)	200	Good	HIV negative	No
19	33	2004	No	585	Poor	Don’t know	Yes
20	39	2003	No	254	Fair	HIV positive	Yes
21	37	2004	No	361	Good	No partner	No partner
22	24	2002	Yes (Jan 2006)	560	Poor	HIV positive	Yes
23	41	2003	Yes (Jan 2009)	Don’t know	Good	HIV positive	Yes
24	34	2002	Yes (May 2009)	214	Good	Don’t know	Yes

Three lay counsellors, with previous counselling experience and training in the Amagugu intervention, delivered the intervention over a six to eight week period. Ethics permission was granted by the Biomedical Research Ethics Committee of the University of KwaZulu-Natal. Written informed consent was obtained from each woman.

### Statistical analysis

Data were entered into a specifically designed Microsoft Access database. Quantitative analyses were carried out using Stata (Version 11.2; Stata Corporation, USA); qualitative data were transcribed, translated, extensively reviewed and categorised by common thematic area.

## Results

All 24 mothers approached agreed to participate, and all completed the intervention. Most women required only one session of each of the visits, however two women repeated Visit 2 (where the mother relates her life and HIV stories) on the suggestion of the counsellor who felt they needed more counselling to work through personal issues before engaging in the intervention training. One mother repeated Visit 4 (where the mother practises the intervention tools she will use with her child), because she requested further opportunities to practise with the counsellor to build her confidence. Seventeen women were literate in both English and Zulu, six were literate in Zulu only and one participant was illiterate. The illiterate participant expressed no reservations about proceeding with the intervention and elicited assistance from a literate family member to assist with reading where required.

Data relating to self-reported health status of mothers is shown in Table 2. The mean age of mothers was 33.5 (median 32; range 24–46) years, most women were living healthily with CD4 counts above 300 (mean 339; median 361; range 1–747) while 9 women had initiated ART (current eligibility for ART in South Africa is a CD4 count of <350 cells/ml). Of the 24 women, 20 reported being unemployed; six received a regular remittance; 23 received a government child support grant; and 11 reported that the child’s father was financially contributing towards the study child's care. Most (19) women reported being unmarried but in a relationship with a long-term partner. In 18 cases the father of the study child was still alive, and eight of the mothers were still in a relationship and living with him. A further two were living with new partners while 14 were living on their own with their children and/or other family members. Fifteen of the 24 women had disclosed their HIV status to their current partner, 12 to a parent and a sibling and 10 to adult friends outside the household. Only three had not disclosed to any other adult, all three of whom were living with a new partner who was unaware of their HIV status. None of these three women had initiated ART and their last CD4 counts were 568, 380 and 200 cells/ml respectively.

The mean age of the 24 children was 6.8 (median 7; range 6–8) years, with a similar numbers of males (13) and females (11) (Table 3). Three children were currently in Grade R (pre-school year); 11 in Grade 1 (first year primary school); seven in Grade 2 (second year primary school); two in Grade 3 (third year primary school); data were missing for one child.

**Table 3:** Maternal disclosure to HIV-negative children

**ID**	**Sex**	**Age**	**Prior disclosure**	**Intervention disclosure**	**Mother report of child's reaction to disclosure**	**Mother reported questions asked by child following disclosure**	**Mother reported what she enjoyed most about disclosure**
01	M	6	No	Virus	Confused	How did you get infected; I haven't seen you go to clinic, when do you go to clinic? What do the nurses and doctors do at clinic?	I really enjoyed the questions the child asked me
02	M	7	No	HIV	Frightened Emotional	Will I get HIV; how can I be safe from HIV?	I enjoyed the peace that I felt after
03	F	6	No	HIV	Confused	What is HIV and what should you be doing at clinic about it?	I enjoyed the materials, because they are so positive about me, even when I am getting sick, I can give them love, the messages are good
04	F	6	No	Virus	Calm	Does a person with this virus become very slim?	I enjoyed that she seemed to understand, like she understands that a person with HIV does not have AIDS
05	F	8	No	Virus	Confused	How did you get the infection? When can I go to clinic?	I enjoyed that the child asked questions I could answer
06	M	8	No	Virus	Calm	No question	I enjoyed playing cards with the child
07	F	7	No	HIV	Confused	Is this what caused my dad to die?; is this why we can’t visit dad's family anymore?	I enjoyed most going through our family life line
08	M	6	Yes	HIV	Calm	No question	I enjoyed every moment of it and that he is the first of my children to know the truth, and I know he is going to come back and ask me about the father soon, then it will all be open
09	M	7	No	HIV	Confused	What is that lion doing in the house with the people in the storybook?	The joy that the storybook brought for the child is what I most enjoyed and I enjoyed being able to give a deep explanation of HIV
10	F	8	No	HIV	Calm	No question	I enjoyed that my child learnt that if a person is sick they will get help from the clinic
11	M	7	No	HIV	Calm	Are you going to die; Is HIV curable?	I enjoyed that I was the one who was able to teach him and that he understood it all
12	M	7	No	HIV	Calm	No question	The fact that my child understood and we had a chance to talk
13	F	6	No	HIV	Calm	No question	I enjoyed all the disclosure steps, they impressed me a lot
14	F	6	No	HIV	Calm	Why do you have to take pills always?	I enjoyed all the steps and I feel more confident to talk about my illness now
15	F	8	Yes	HIV	Calm	Is this what happened to my father? Did you love my father? How did you get the HIV, did you get it from my father or another man? Would my father be alive if he took medicine?	I really enjoyed everything about doing disclosure
16	M	7	No	Virus	Calm Frightened	No question	I enjoyed the whole thing from the beginning to the end
17	M	8	No	Virus	Confused	Do all viruses kill people? Are you going to die from the virus? Will I be left with no parents?	I enjoyed that my child was interested and listened to me
18	M	7	No	Virus	Calm	Does my niece who takes pills have this Virus also?	Telling him was important for him to know, it was exciting and I felt good, I felt at peace for no longer keeping it from him
19	M	6	No	Virus	Calm Confused	Do I have a virus now? Should I eat more vegetables?	I enjoyed our talk, and it brought back hope to me as I was talking to him
20	M	6	Yes	Virus*	Calm	What is AIDS, is it the same as a virus? Does my stepfather have it? Did my father have it before he died; Are ARVs the same as the tablets you showed me on the body map? Does AIDS ever get finished in the body?	I enjoyed and I like the fact that he was so quick to understand, he is clever
21	F	6	No	Virus*	Calm	What is this virus called?	I enjoyed using all the materials
22	M	7	No	Virus	Calm	No question	I enjoyed most that my child understood most of what we did, I wasn't expecting it
23	F	7	Yes	Virus	Calm Confused	No question	I enjoyed having a chance to talk about my status
24	F	7	Yes	Virus	Calm	No question	I enjoy the materials because of the good messages it brings to me and my children

*In cases where mother undertook disclosure using words Virus and child’s questions led to a discussion on HIV or AIDS using those words, disclosure is listed as partial given that mother-led disclosure was initially partial.

Five mothers indicated that they had either ‘partially or fully disclosed’ to their children prior to this intervention, mostly driven by pragmatic reasons following an illness and a need to ensure child care. In all five cases the mothers had fully disclosed (i.e. used the words “HIV”) to their older children (10–18 years), but had told their younger children (<10 years) that they had an illness which required them to go to hospital. While HIV was spoken about with older children and other family members, and it was possible the younger children were aware of, or had heard about HIV, in the family, in these five cases the mothers were unsure what the younger child knew and understood about HIV at baseline. As a result, all five of these mothers wished to participate in this intervention to disclose fully to the younger index child, as they felt their previous partial disclosures around illness and hospitalisations had been inadequate. These mothers were considered to have a clear intention to disclose at the start of the intervention. Of the remaining 19 women, three had disclosed fully to an older child but had made no disclosure to the younger index child while 16 had not disclosed to any of their children regardless of their age. None of these 19 mothers expressed any intention to disclose to the index child prior to enrolling in the study.

The most salient concerns about disclosure prior to the disclosure intervention were that the child was too young (22/24), might suffer emotionally afterwards (21/24), would disclose to others (21/24) or that the mother felt unable to answer difficult questions relating to the source of the HIV infection or death (19/24).

All 24 mothers chose to disclose something about their illness to their children during the intervention, but levels of HIV disclosure differed. Almost half the mothers (11) disclosed fully using the words “HIV” and indicating to the child that they were infected with HIV, while the remaining 13 disclosed partially, explaining that they had a virus, and using the tools to explain what the virus did in their bodies and how it can be controlled, without specifically using the words “HIV”. Among the 19 mothers who had not disclosed previously, and had no intention to disclose at the start of the intervention, 8/19 fully disclosed using the words ‘HIV’ while 11/19 chose to undertake partial disclosure. In two cases indicated by *in Table 3 (Family Study Numbers 20 and 21), the mother disclosed using the words "Virus" during the disclosure activity, but the child subsequently began asking questions about “AIDS” in the case of Family 20 and directly asked for the virus name in the case of Family 21. In Family 21 the mother did not name “HIV” while in Family 20 the mother shifted to discussing HIV openly. In both these cases the maternal disclosure is listed as partial as this most closely reflects the mothers’ intention and action during the intervention disclosure. However, in these two cases it is clear that for one child (Family 20) significant knowledge of HIV may have preceded the intervention, and for the other (Family 21) when the mother was presented with an option to disclose more fully she continued with partial disclosure. It is noteworthy that of the 5/11 mothers who stated they had disclosed fully to older children prior to the intervention and intended to disclose fully to younger children as part of the intervention, only 2/5 subsequently disclosed fully and reported their children were calm (Family 8 and 15); the remaining 3/5 chose to disclose partially. Unlike in Family 20, in Families 23 and 24 the children’s questions following partial disclosure did not indicate any knowledge of HIV or willingness to raise questions about HIV in response to the partial disclosure.

Most mothers described the reaction of their children to the disclosure intervention as calm, accepting and confident (14), eight reported that their child was initially confused and in need of further explanation, and only two reported fear or emotional distress as the initial reac-tion to disclosure. Table 3 summarises children’s reactions, commonly asked questions after disclosure and mothers’ experiences of undertaking disclosure using the intervention materials. A few mothers (6/24) reported that there were aspects of disclosure that they did not enjoy, including: feeling frustrated that the child did not understand initially, feeling disappointed that the child had not asked more questions, feeling concerned that knowing about her HIV status was hurtful for the child and caused emotional distress, finding it difficult to talk openly about HIV, and feeling nervous about using the HIV Body Map and saying the words “HIV” out loud. Two mothers reported not enjoying the Family Life Line exercise (Session 4, Table 1), the first because it brought up difficult issues around step-siblings in the household who were raised by the current partner, and the other because it raised difficult issues about bereavement and people who had died which she felt ill-prepared for. The most salient challenge raised by mothers in the post-disclosure interview was finding a quiet space in the home to undertake disclosure, with many mothers reporting having to deal with interruptions by younger children who wanted to play with the intervention materials.

While family members were encouraged to participate in the intervention, only a few (3/24) helped the mother with practice and preparation, two of whom subsequently participated in the disclosure event with the mother. However, more family members became involved in health promotion and custody planning activities following the disclosure being completed by the mother. Four mothers reported challenges in taking their children to clinic as they were employed: in two cases the father of the child completed the clinic component of the intervention, and in the remaining two an older sibling accompanied the child to the clinic.

### Mothers perceptions of the intervention and materials

Prior to the intervention mothers expressed concerns about disclosure including fears that the child might raise issues about HIV and death:concerns that the child was too young to understand:worries that the disclosure would cause the child emotional damage:and fears that the child would tell others about her HIV status:

“They can be full of fear that I may die anytime soon”

“She is still too young and not clever enough to understand”

“I felt that they would not accept it, they would get frustrated and not do well at school”

“My child talks too much, she may tell other people”

However, the materials, illustrations and branding reinforced maternal confidence to disclose their HIV status, improving maternal self-efficacy:providing confidence around the quality of the materials:making the disclosure process seem more realistic and approachable:helping the mother to organise her thoughts in a structured way:and giving a structure for explaining health related issues:

“I was so nervous about the telling, saying the words, but now I see how the body map works, I can just show the child and take myself there easy”

I feel like this is going to be very useful in showing me how to do it well for my child

“The children will feel too excited because it looks like it came from the shops”

“These materials makes disclosure less scary for me”

“The steps are easy to understand, in the way that they are presented, I will enjoy to use it, I can’t easily get lost”

“I really like the materials, I have been struggling to explain to my children about clinic, why I take the pills, and they have not understood clearly, with these materials even I understand more clearly”

This body map makes a real contribution I can use it to explain so many things from HIV to even the flu

For most mothers in the intervention, the materials (see Table 1) were the first of their kind in the household. Only seven households reported having any storybook in the household prior to the intervention and 22 reported that the story book helped them to spend more time with the child. All women expressed the desire for access to similar books covering topics including: Teaching children to be safe from child sexual abuse; Teaching young children about their bodies and sex education; Teaching morals and values and good behaviour. Similarly, only nine mothers reported that any of their children had a doll prior to the intervention, and most felt it was a useful ‘play and communication’ tool, reminding them to play with their child (18), helping them to listen to their child (19) and understand their child’s worries better (18), and recognising what makes their child feel happy and reassured (17).

#### Counsellors’ perceptions of the intervention and materials

An important aspect of the intervention development was ensuring that the structure of the materials assisted the counsellor to focus on the mothers' emotional issues. The counsellors reported finding the tools useful in containing emotions and organising narratives, and maintaining counsellor empathy with the mother:

“*It was so simple now that we are doing it, the different steps you do, first it is listening and it’s even a surprise to the person them self…they think you just want to know about them being HIV-infected..then they are surprised you actually want to know their love stories, it’s a good surprise, everything about their face and body changes, because they see I am looking at them and that I see them and not the HIV positive label”* [Counsellor 1]

*“It helps that we talk and then do a timeline, it helps to bring the story together, to make sense of things, it becomes that you are looking at it together, so you start to feel you get a same view, like you are both looking out the same window”* [Counsellor 2]

*“Asking mothers to tell their love stories as part of their life stories, well it’s different to what I expected, you learn to be humble about yourself, we all make mistakes in love, you don’t have to make many or even big mistakes before you can get HIV, when you take people back in time they have a chance to explain, you are saying to them that you are there to help, you are a real person who knows the real world, and you want to deal with real life”* [Counsellor 3]

Quality assurance (QA) visits were conducted by a masters level project coordinator with a randomly selected sample of 10% of visits and counsellors scored >80% on all QA visits for all sessions. Each session included a rating scale covering core concepts and step fidelity; raw scores were converted to percentages and averaged over sessions by counsellor. Failures on QA most commonly related to fidelity in the order, rather than omission, of steps in particular intervention sessions. On average, the individual and family preparation phase of the intervention required 1–2 counsellor hours (Session 1 and 2), disclosure training took 2–3 hours (Session 3 and 4), health promotion training 1–2 hours (Session 5). Mothers reported that on average the disclosure process took less than one hour with the child; all mothers completed the intervention within an 8 week period.

#### Community perceptions of the intervention and materials

Finally, ensuring cultural sensitivity in the development of the illustrations was important in the piloting of the materials:

*“… to see those illustrations and the stories and stuff, I mean, you know, as a Zulu person out there in the world you just don’t see that happening, to have something that looks like you, not like a cartoon with black coloured skin or those stupid things you know, but that really looks like you, your culture, the colours, your hair; the way people dress, you just don’t see things like that, you don’t really even think that you haven’t seen them until you see them and then you say ‘hey look at this – this is really cool, why haven’t I seen this before now, I’m a Zulu person and this looks like me, this looks like my family, you know what I mean, it’s really cool and it counts for a lot, and it says something about who you are and how you want to help and respect people in this community.”* [Member of the Africa Centre Community Liaison Office]

### Children’s reactions to disclosure

After the disclosure, the questions children asked of their mothers were similar to those commonly cited in the literature, relating mainly to the nature of HIV, transmission and HIV medication (Table 3). Several children sought reassurance that they were not infected themselves and that the mother was not going to die soon. An unusual finding related to the level of inadvertent disclosure of siblings' and other adults' HIV status that occurred as a result of maternal disclosure and discussion of HIV-related issues (see children’s questions in Table 3).

### The intervention impact on family relationships, health promotion and care planning

As shown in Table 4, mothers perceived that this intervention resulted in improvements in family relationships and understanding of roles and responsibilities towards child care, increased health promotion activities in the homestead and improved parent confidence around health promotion and sex education, increased discussion around child protection risks including bullying, teacher problems, physical and sexual abuse. At the post-intervention interview, 6 of the 24 mothers reported that, as a result of the intervention, they had disclosed to an adult family member whom they had not disclosed to previously, suggesting that the intervention may increase disclosure more generally in the family. While the intervention resulted in these changes within the home environment and family relationship, the same increases in confidence were not evidenced in maternal confidence to effect change in their broader community.

**Table 4:** Intervention impact on family support, health promotion, maternal confidence and child protection (Questions asked to the mother)

**Family support**			
**As a result of the family support activities do you believe:**	**Mothers agree**	**Mothers disagree**	**Don't know**
The child is able to identify who can support and care for them in the family (more than in the past)	20	2	2
The family feels it is clear that they are responsible for the care of the child (more than in the past)	21	0	3
I feel more supported and cared for in my role as a parent by my family members	22	0	2
I feel less anxious about the future care of my child or children if something happens to me	22	0	2
**Health promotion**			
**Following the intervention have you continued to use the materials**	**Not at all**	**A few times**	**Everyday**
In the last week how many times have you or your child used the body map with anybody in your family?	7	11	6
In the last week how many times have you or your child used the health promotion playing cards with anybody in your family?	13	10	1
**Thinking about the body map and how it was used in this intervention:**	**Yes**	**No**	**Don't know**
Could you use it to teach about other health issues in the future	21	2	1
Could you use it for sex education in the future	21	0	3
**Maternal confidence**			
**Given what you have learnt in this intervention, would you feel confident to:**	**Yes**	**No**	**Don't know**
Help other HIV positive mothers in your community to talk about disclosure with their children	8	2	14
Help community stakeholders understand how to talk about disclosure with their children	9	0	15
Advocate for yourself and your child’s health rights at the clinic and share what you know about disclosure	9	0	15
**Child protection**			
**Before this intervention, had you or anyone in your family ever talked with your child about:**	**Yes**	**No**	**Missing**
Risks of bullying from friends	11	13	0
Teacher-child problems	8	16	0
Physical abuse	10	14	0
Sexual abuse	7	17	0
**As a result of the intervention did you talk with your child about:**			
Risks of bullying from friends	19	3	2
Teacher-child problems	19	3	2
Physical abuse	18	4	1
Sexual abuse	14	8	2

## Discussion

This pilot study with 24 families showed high levels of acceptability and encouraging results in terms of effectiveness as defined by mothers undertaking full or partial disclosure. Almost half the mothers who had never previously disclosed to a child in the family, disclosed to a child aged 6–10 years making a full disclosure using the words ‘HIV’ as part of this intervention. Participation and disclosure rates suggest the intervention package is culturally acceptable and there appear to be no major barriers to intervention support using this family-centred model. Thus, the intervention shows promise for supporting maternal HIV disclosure in rural HIV endemic settings in Southern Africa.

Our data suggest that decisions around disclosure are complex, and the choice to make a full or partial disclosure may be dependent on several factors. The small sample size in this pilot prevents any meaningful analysis of factors associated with the choice to make a full or partial disclosure, for example child age or gender.

It is possible that the younger age of most children in this sample may have influenced mothers’ preference for partial disclosure, but it is also possible that the preference for partial disclosure was influenced by other factors such as the mothers’ personal readiness or household factors not captured in the pilot. Further research is required to better understand these decisions and how disclosure intentions of mothers prior to the intervention influence whether or how much they disclose to their children.

While these findings are preliminary and should be interpreted with caution, they are encouraging. The intervention is innovative in that it targets a young age group in a high HIV prevalence area. To our knowledge this is the first intervention targeting primary school-age children (6–10 years) from a resource-limited setting, with the intervention messages focusing not only on HIV disclosure itself but also on health promotion for pre-adolescent children. As in the TRACK program [28], we focus on the ‘relationship context’ and improving parenting skills [68], which have been associated with decreased child problem behaviours [28,53,55]. However, our intervention is somewhat more intensive, involving six as opposed to three home visits, is more directive and supplemented in each session with illustrated materials and games to help mothers facilitate disclosure and to assist lay counsellors to focus on the topic. This increased intensity, structure and the strength of the counselling relationship may explain the positive disclosure rates. It is not appropriate to compare these results to the TRACK program which reported post intervention disclosure rates of 33% in the intervention group and 7% in the control given that the TRACK program only enrolled mothers with no intention to disclose. However, in light of generally low disclosure rates around the world, achieving close to fifty percent full HIV disclosure in this pilot study, using a locally adapted, similar approach to other international studies shows promise for future work in this area. Further research is required to test whether these findings are replicable and the intervention feasible at a larger scale. A larger demonstration project which aims to enrol 300 families is currently underway.

While the intervention was highly directive and structured, feedback from mothers and counsellors suggests that an important intervention component was the focus on counselling, and training the mother to achieve competence to intervene with her own children as opposed to study counsellors directly intervening with children. This was highly acceptable to both counsellors and mothers and likely accounts for reported improvements in maternal confidence to deal with HIV and health-related matters in the family.

Importantly, this intervention was conducted by lay counsellors, who were able to learn to use the materials and deliver the intervention with this pilot group of mothers. This is particularly important for later scale-up given the increasing time constraints on professional health staff within HIV programmes. Task shifting is already taking place in many areas of health care in sub-Saharan Africa, with lay counsellors or CHWs taking on roles in infant feeding counselling, HIV counselling, ART adherence, and nutrition counselling [69,70]. Lay counsellors and CHWs have shown ability to conduct psycho-social interventions at the primary health care level, including HIV counselling, testing, and training to initiate HIV treatment [71] and to deliver simplified psychological interventions in poorly resourced settings [72,73]. Further, the South African National Department of Health plans to include community health care workers as key players in the new primary health care teams, which will focus particularly on improving maternal and child health outcomes [61].

However, little intervention research has focused on the development of techniques, activities and guidelines to train lay counsellors or CHWs to deliver psychologically complex interventions in Southern Africa. To address this, our intervention approach considered several common factors known to impact on therapeutic outcomes [74]. The intervention specifically focused on integrating structured counsellor activities to increase counsellor empathy, positive unconditional regard, and to facilitate encouragement and acknowledgment of achievements by the mother. Such factors are known as ‘therapeutic variables’, and have been shown to account for at least a third of positive outcomes in psychotherapy [75] and social work [76]. It is possible that a similar intervention approach could be applied to a variety of topic areas addressing challenges that HIV-positive parents and families face.

Besides ‘therapeutic variables’ we considered ‘extra therapeutic variables’ known to contribute to positive outcomes in therapeutic relationships, including increasing maternal support in the home and the health care system [75]. The intervention successfully draws on and adapts established therapeutic techniques and processes (from psychotherapy, family and cognitive behavioural therapy) into a structured intervention which facilitates the enactment of therapeu-tic techniques by a lay counsellor. Counsellors found the materials user-friendly, and quality assurance data suggests that the materials assisted in ensuring a high level of intervention fidelity, which has been shown to be a challenge in family HIV interventions [35].

In line with the international literature [7,33], the qualitative data suggests that mothers had significant distress around disclosure of their own HIV status to their children and felt unable to approach the topic without intervention support. In particular, the qualitative data from participating mothers suggests that these investments in material design and culturally-sensitive illustrations have proved critical to reinforcing maternal self-efficacy. Investing in high quality, age-appropriate and attractively designed materials communicates value and respect, reinforces positive attributes and inspires confidence in HIV disclosure, helping the mother and counsellor to organise their thoughts around the disclosure process. Children’s questions were fairly predictable and the counsellor and mother had worked through these questions and some possible ways to answer them in Visit 4.

## Conclusion

In summary this intervention illustrates that complex therapeutic processes can be transformed into practical, structured, step-by-step activities that encourage both experiential learning and reflective practice, and bring about health behaviour change at a familial level. There are several limitations to this study, most importantly it is a small pilot study, and the sample size does not allow for wide generalisation of the findings. Furthermore, given the convenience sampling strategy and lack of a control group it is possible that the results are influenced by selection bias. Further research is required to test these results in a larger sample and a controlled study, to determine the factors that influence the degree of maternal disclosure, and the impact this disclosure has for mothers, children and families over time.

## Endnotes

^a^Amagugu is a Zulu term meaning “Treasures” to capture the concept that children are our future and are precious.

^b^For information on play for communication see http://www.dlalanathi.org.za and http://www.uthandoproject.org.

## Competing interests

We declare no competing interests. This study was funded by the Canadian International Development Agency (CIDA). Ruth Bland and Tamsen Rochat received salary support from the Wellcome Trust-funded Africa Centre. Tamsen Rochat and Ntombizodumo Mkwanazi also received salary support from CIDA.

## Authors’ contributions

TR contributed to the design and development of the intervention, assisted in securing funding and ethical permissions for the research, provided oversight of data collection and data entry and was responsible for data analysis and interpretation, and the initial draft and further revisions of the manuscript. NM participated in the development of intervention materials and the training and oversight of intervention staff; she was responsible for the collection and entry of data during the pilot study and assisted with data cleaning and interpretation of the results. She assisted with literature searches and referencing on the initial draft of the manuscript and contributed to critical revisions of the revised manuscript. RB led the design and development of the intervention, was responsible for securing funding and ethical permissions for the research, provided oversight of data analysis and interpretation of results, and provided significant input into both the initial draft and further critical revisions of the manuscript. All authors read and approved the final manuscript.

## Pre-publication history

The pre-publication history for this paper can be accessed here:

http://www.biomedcentral.com/1471-2458/13/147/prepub
